# Proton Pump Inhibitor‐Induced Fundic Gland Polyps With Massive Bleeding Regressed on Alternative Histamine 2 Receptor Antagonist Therapy

**DOI:** 10.1002/deo2.70273

**Published:** 2026-01-15

**Authors:** Ryosuke Ikeda, Hiroaki Kaneko, Hiroki Sato, Yuto Matsuoka, Tomomi Hamaguchi, Aya Ikeda, Yoshihiro Goda, Soichiro Sue, Kuniyasu Irie, Shin Maeda

**Affiliations:** ^1^ Department of Gastroenterology Yokohama City University Graduate School of Medicine Yokohama Japan

**Keywords:** anticoagulant agent, gastrointestinal bleeding, histamine 2 receptor antagonist, polyp regression, proton pump inhibitor‐induced fundic gland polyp

## Abstract

We report a case of massive bleeding from proton pump inhibitor (PPI)‐induced fundic gland polyps (FGPs) that regressed after switching to a histamine‐2 receptor antagonist (H2RA). A 46‐year‐old man with antiphospholipid syndrome had been receiving warfarin and lansoprazole for 4 years. Esophagogastroduodenoscopy (EGD) revealed multiple enlarged, edematous FGPs compared to those observed 3 years earlier. One month later, the patient presented with melena, anemia, and transient loss of consciousness. Laboratory data revealed anemia and a prolonged prothrombin time/international normalized ratio (PT‐INR). Emergency EGD showed refractory oozing from the FGPs caused by insufflation and water jet stimulation. The bleeding was successfully controlled with vitamin K administration. After PT‐INR normalization, no further bleeding occurred, and a follow‐up EGD 3 days later showed no bleeding recurrence. We considered that PPI therapy might lead to recurrent bleeding from the FGPs and switched therapy to an H2RA. Follow‐up EGD at 2 and 6 months revealed gradual and marked regression of the FGPs. This case demonstrates that PPI‐induced FGPs can result in massive bleeding, particularly in patients receiving anticoagulant therapy. Furthermore, FGP regression following the switch to H2RA suggests that H2RA therapy may be an alternative treatment when discontinuation of PPI therapy is not feasible.

## 1 Introduction

Proton pump inhibitors (PPIs) are widely prescribed acid‐suppressive agents for the management of peptic ulcers and gastroesophageal reflux disease. In recent years, several studies have reported that long‐term PPI therapy can lead to an increased prevalence and enlargement of fundic gland polyps (FGPs) [1, 2]. While most PPI‐induced FGPs are benign and asymptomatic, mild bleeding has occasionally been reported [3, 4]. We report a case of massive bleeding arising from PPI‐induced FGPs that regressed after switching to a histamine‐2 receptor antagonist (H2RA).

## 2 Case Report

**FIGURE 1:**
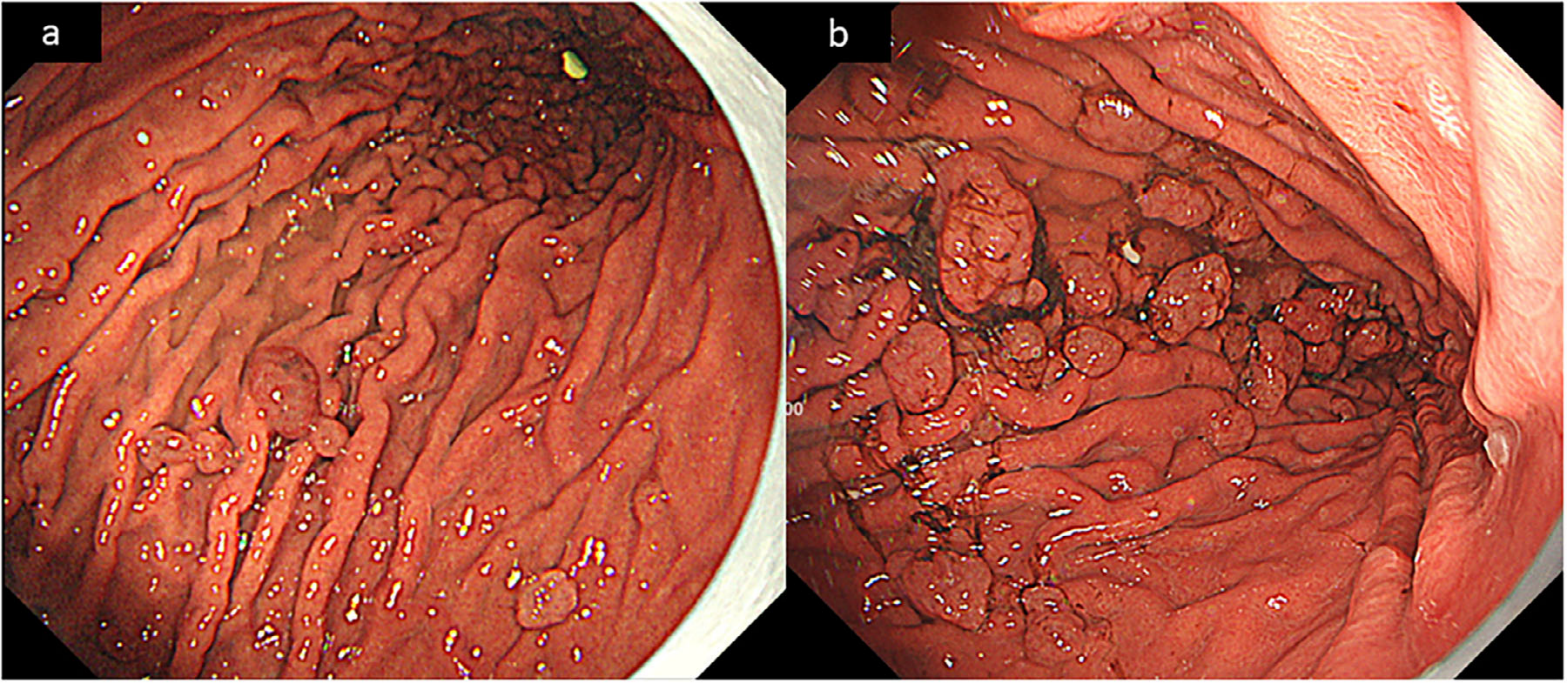
Endoscopic images showing sequential changes of FGPs. (a) Endoscopic findings 3 years before emergency presentation: white‐light imaging revealed slightly edematous fundic gland polyps (FGPs) with mild edematous changes. (b) Endoscopic findings at the present examination: the FGPs were clearly enlarged and increased in number compared with those observed 3 years earlier, and slight blood attachment was observed on the surface of several polyps.

A 46‐year‐old man with a history of antiphospholipid syndrome had been receiving oral warfarin therapy for the prevention of thromboembolic events for 4 years, with a prothrombin time‐international normalized ratio (PT‐INR) maintained between 2 and 3. The patient had also been taking 15 mg of lansoprazole daily to prevent upper gastrointestinal bleeding. Esophagogastroduodenoscopy (EGD) for routine surveillance revealed multiple enlarged FGPs compared with 3 years earlier (Figure 1). The polyps appeared markedly edematous, with an increase in both number and size. There was no active bleeding; however, a slight attachment of blood was observed on the surface of the polyps. After long‐term PPI therapy and progressive polyp enlargement, with lesions showing a color similar to the surrounding mucosa and visible dilated vessels on the surface, we endoscopically diagnosed the patient with PPI‐induced FGPs based on endoscopic findings.

**FIGURE 2:**
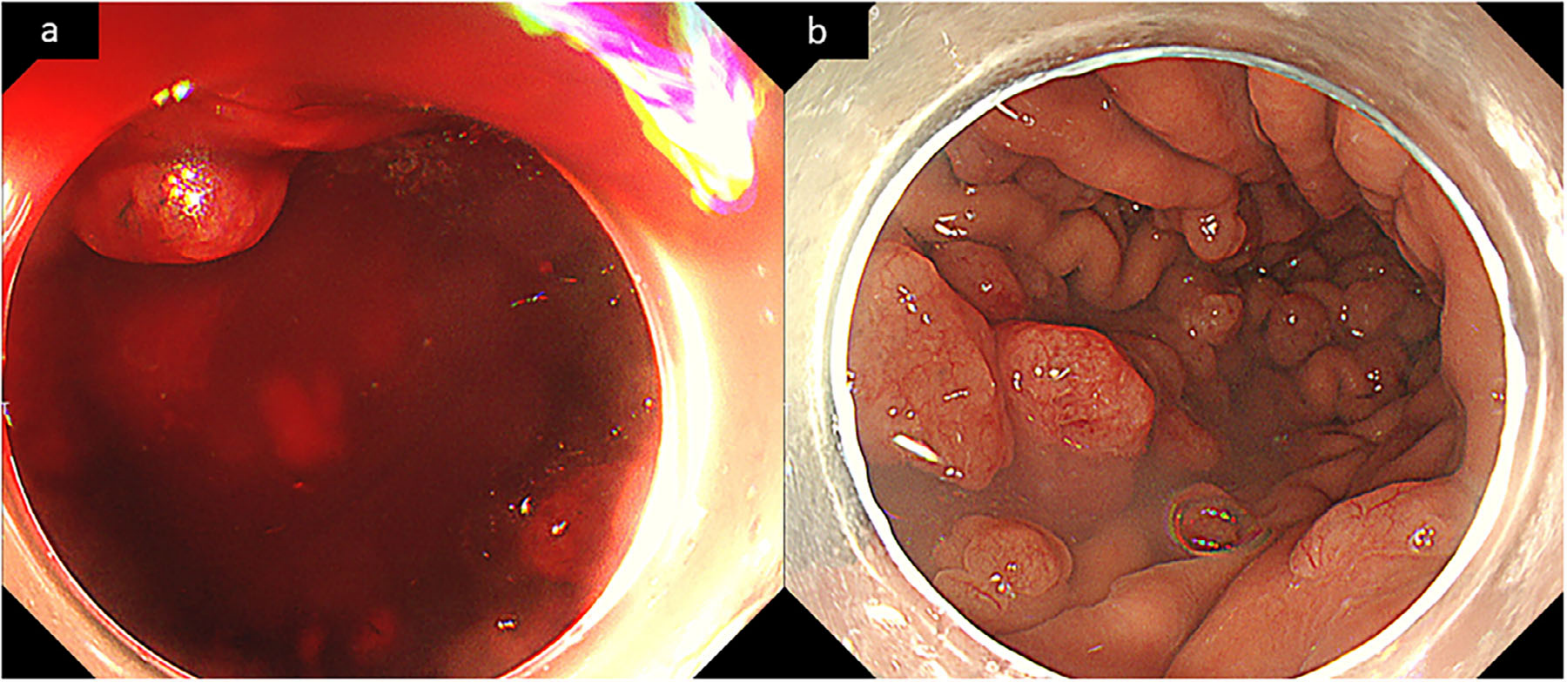
Endoscopic images at the time of massive bleeding. (a) At initial endoscope insertion, no blood was observed; however, stimulation by insufflation and a water jet induced refractory oozing of the fundic gland polyps (FGPs). (b) Endoscopic findings 3 days after emergency endoscopy: Enlarged FGPs were still present, but no bleeding tendency was observed upon stimulation, and no other lesions that could account for bleeding were observed.

One month after the endoscopic examination, the patient presented with melena, symptoms of anemia, and transient loss of consciousness due to hypotension and underwent emergency admission. Laboratory tests revealed a hemoglobin level of 10.4 g/dL, a decrease from his previous baseline of 14–15 g/dL, and a markedly prolonged PT‐INR of 3.37 (Table 1). Emergency EGD was performed for suspected upper gastrointestinal bleeding. At initial endoscope insertion, no active bleeding was observed; however, stimulation by insufflation and the water jet induced refractory oozing from the FGPs (Figure 2). As no arterial bleeding was observed, topical thrombin (Thrombin Granules; Sawai Pharmaceutical Co., Ltd., Osaka, Japan) was applied directly onto the surface of the polyps, and vitamin K was administered to reverse the PT‐INR prolongation, along with red blood cell transfusions. Thereafter, there was no persistent bleeding, and the PT‐INR was within the normal range. On follow‐up EGD performed 3 days later, the multiple edematous FGPs showed no bleeding tendency, even upon stimulation, and no other lesions that could account for the bleeding were observed.

**TABLE 1:** Laboratory data on admission.

Hematology					
**Complete blood cell counts**			**Biochemistry**		
WBC	10.7	×10^3^/µL	TP	5.8	g/dL
RBC	355	×10^4^/µL	Alb	3.7	g/dL
Hb	10.4	g/dL	CK	81	U/L
Hct	30.6	%	AST	12	U/L
Plt	23.7	×10^4^/µL	ALT	12	U/L
**Coagulation**			LDH	115	U/L
APTT	36.9	sec	ALP	50	U/L
PT‐INR	3.36	ratio	γ‐GTP	15	U/L
Fib	206	mg/dL	AMY	414	U/L
D‐dimer	<0.50	µg/mL	Cr	1.01	mg/dL
			BUN	43	mg/dL
			Na	140	mmol/L
			K	3.8	mmol/L
			Cl	108	mmol/L
			CRP	0.07	mg/dL

Abbreviations: γ‐GTP: gamma‐glutamyl transpeptidase, Alb: albumin, ALP: alkaline phosphatase, ALT: alanine aminotransferase, AMY: amylase, APTT: activated partial thromboplastin time, AST: aspartate aminotransferase, BUN: blood urea nitrogen, Cl: chloride, CK: creatine kinase, Cr: creatinine, CRP: C‐reactive protein, Fib: fibrinogen, Hb: hemoglobin, Hct: hematocrit, K: potassium, LDH: lactate dehydrogenase, Na: sodium, Plt: platelet, PT‐INR: prothrombin time international normalized ratio, RBC: red blood cell, TP: total protein, WBC: white blood cell.

**FIGURE 3:**
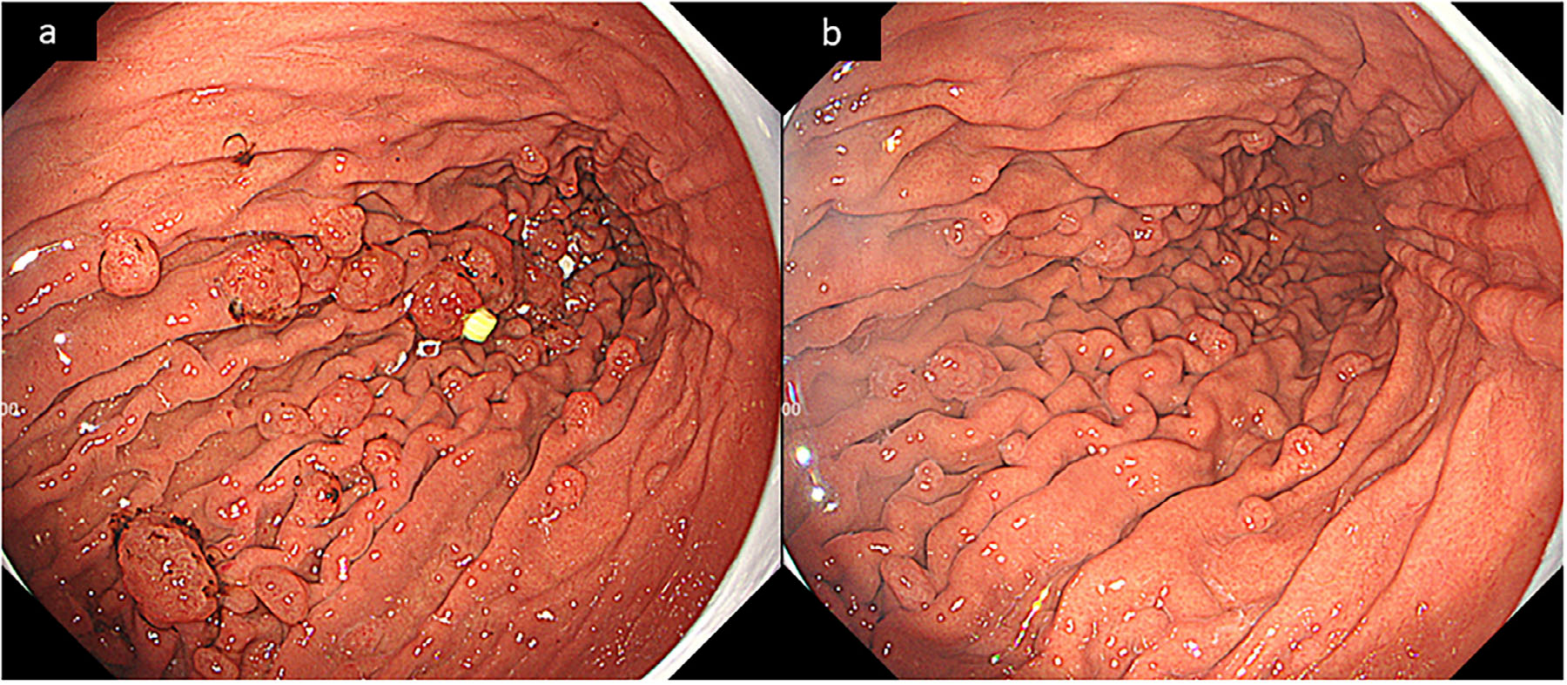
Endoscopic images after switching to an H2RA. (a) Endoscopic findings 2 months after switching to a histamine‐2 receptor antagonist (H2RA): The edematous fundic gland polyps (FGPs) persisted, a mild reduction in polyp height and number was observed, and surface blood attachment had disappeared. (b) Endoscopic findings 6 months after switching to an H2RA: The FGPs showed marked regression, with a clear decrease in both polyp size and overall number compared with those at the time of H2RA initiation.

When considering that the PPI therapy might have led to recurrent bleeding from the FGPs, we switched therapy to an H2RA and adjusted the warfarin dose to maintain the PT‐INR at a relatively low range of approximately 2. The patient was switched to famotidine at a dose of 20 mg twice daily; no reflux‐related symptoms such as heartburn, regurgitation, or epigastric pain were observed after the switch, and no additional rescue acid‐suppressive therapy was required. At 2 months after switching to H2RA, the polyps showed a mild reduction in height and number, with the disappearance of surface blood attachment (Figure 3), while at 6 months, both the number and size of FGPs were markedly decreased compared with those at the time of H2RA initiation.

## 3 Discussion

We report a rare case of PPI‐induced FGPs with massive bleeding that subsequently regressed following a switch to an H2RA. FGPs are commonly detected benign lesions defined by cystic dilatation of the fundic glands. Several studies have suggested an association between long‐term PPI therapy and FGPs. Patients receiving PPIs show a significantly higher prevalence of FGPs than non‐PPI users, and the prevalence increases with longer therapy duration, suggesting a time‐dependent relationship [1]. Moreover, PPI users tend to develop FGPs that are larger in size and greater in number than non‐PPI users or H2RA users [2].

Several studies have investigated the mechanisms underlying PPI‐induced FGP development and enlargement. Synnerstad et al. examined the gastric mucosa of rats and demonstrated that omeprazole administration significantly increased intraglandular pressure, leading to fluid retention, ductal dilatation, and subsequent polyp growth [5]. In addition, Cats et al. analyzed gastric biopsies from patients with gastroesophageal reflux disease treated with omeprazole for 1 year [6]. They reported a high frequency of parietal cell protrusion (PCP) during long‐term PPI use and proposed that PCP could cause glandular obstruction, resulting in fundic gland cysts and FGP formation. In our case, the patient also had a 4‐year history of PPI administration, and it is reasonable to assume that a similar mechanism of cystic dilatation contributed to polyp enlargement. Although a pathological evaluation was not performed because biopsy or endoscopic resection posed a high risk of bleeding in this patient, this mechanism is considered plausible.

Complications of PPI‐induced FGPs include malignant transformation and secondary disorders related to polyp enlargement. FGPs associated with familial adenomatous polyposis have frequently been reported to show dysplasia or malignant transformation. In contrast, malignant transformation in sporadic FGPs, as in our case, is extremely rare, estimated to occur in approximately 1% of polyps [7]. Intussusception and bleeding are secondary disorders associated with tumor enlargement. In cases of intussusception, a case has been reported where enlarged FGPs induced by long‐term vonoprazan therapy prolapse into the duodenal bulb, resulting in gastric outlet obstruction [8]. In the case of bleeding, several reports have described multiple FGPs induced by long‐term PPI therapy causing recurrent bleeding, presenting as chronic anemia or melena [3, 4]. However, in most of these reports, the severity was limited to mild bleeding. To the best of our knowledge, no cases of massive bleeding resulting in hemodynamic shock have been reported to date. In the present case, the primary factor contributing to the severity of massive bleeding was the bleeding tendency associated with excessive prolongation of the PT‐INR. However, endoscopic findings prior to bleeding revealed slight blood attachment on the surface of the cystically dilated FGPs. These findings suggest that the enlarged FGPs demonstrated cystic dilatation with thinning and fragility of the superficial mucosa, making them vulnerable to bleeding. Therefore, we consider that the massive bleeding in this patient resulted from the combined effects of warfarin‐related anticoagulation with markedly prolonged PT‐INR and increased mucosal fragility of the edematous and cystically dilated FGPs.

Regarding the treatment of PPI‐induced FGPs, discontinuation of PPI therapy is considered the most effective approach, and multiple reports have documented the regression of FGPs following PPI withdrawal [3, 4, 9]. However, in patients who require PPI therapy for conditions such as gastroesophageal reflux disease or peptic ulcers, complete discontinuation is often difficult. In our case, the patient was taking anticoagulants and had a high risk of bleeding; therefore, discontinuation of PPI therapy was not feasible, and the medication was switched to an H2RA. Subsequently, the polyps regressed gradually. Although only a few reports have described the effectiveness of switching to H2RAs [10], our case further supports this finding.

There is no high‐level evidence clarifying the mechanism of the regression of FGPs with H2RA therapy; however, Shinozaki et al. reported that the prevalence of FGPs was 52.3% in patients receiving PPIs compared with 23.4% in those receiving H2RAs. These findings suggest that the weaker acid‐suppressive effects of H2RAs may reduce excessive glandular obstruction and suppress cystic progression, thereby contributing to polyp regression.

In conclusion, although most PPI‐induced FGPs remain asymptomatic, they may occasionally present with bleeding. Particular attention should be paid to the risk of massive bleeding in patients receiving anticoagulant therapy. Moreover, this case suggests that switching to H2RAs can achieve FGP regression and may represent a viable therapeutic option.

## Author Contributions

**Ikeda Ryosuke** contributed to the conception and designed the study. **Ikeda Ryosuke**, **Kaneko Hiroaki**, **Sato Hiroki**, **Matsuoka Yuto**, **Hamaguchi Tomomi**, **Ikeda Aya**, **Goda Yoshihiro**, **Sue Soichiro**, and **Irie Kuniyasu** were members of a medical team treating the patient. **Ikeda Ryosuke** and **Maeda Shin** contributed to the drafting of the article. **Maeda Shin** contributed to the critical revision of this article. **Maeda Shin** approved the final draft of the manuscript. All the authors listed have contributed substantially to the design and editing of the manuscript.

## Funding

The authors received no specific funding for this work.

## Ethics Statement

All procedures followed were in accordance with the ethical standards of the responsible committee on human experimentation (institutional and national) and with the Helsinki Declaration of 1975, as revised in 2008.

## Consent

Informed consent was obtained from the patient in this case report.

## Conflicts of Interest

The authors declare no conflicts of interest.
